# Effects of body mass index on left ventricular function: An echocardiographic comparison between obese and nonobese individuals

**DOI:** 10.14814/phy2.70705

**Published:** 2025-12-28

**Authors:** Nowrin Kashem, Foysal Ahmed, Sumona Tanu, Anamur Rahman, Rehana Sultana, Yeasmin Jui, Md Asaduzzaman

**Affiliations:** ^1^ Department of Physiology Sylhet MAG Osmani Medical College Sylhet Bangladesh; ^2^ Department of Pharmacology Sylhet MAG Osmani Medical College Sylhet Bangladesh; ^3^ Department of Cardiology Sylhet MAG Osmani Medical College Sylhet Bangladesh; ^4^ Department of Medicine Sylhet MAG Osmani Medical College Hospital Sylhet Bangladesh

**Keywords:** body mass index, diastolic dysfunction, echocardiography, ejection fraction, left ventricular function, obesity

## Abstract

Obesity is linked to structural and functional cardiac changes. This study assessed the impact of body mass index (BMI) on left ventricular (LV) function. A cross‐sectional study included 100 adults (50 obese and 50 nonobese), matched for age and sex. Echocardiographic parameters, end‐diastolic volume (EDV), end‐systolic volume (ESV), ejection fraction (EF), E/A, E/e ratios, Interventricular Septal thickness in Diastole (IVSd), stroke volume (SV), and cardiac output (CO), were measured by a blinded cardiologist using American Society of Echocardiography (guidelines. Spearman's correlation and multivariable regression evaluated associations between BMI and LV parameters. Obese participants had higher EDV (100.0 ± 23.3 vs. 84.6 ± 25.8 mL), ESV (35.6 ± 10.0 vs. 30.4 ± 12.5 mL), IVSd (9.33 ± 1.53 vs. 8.37 ± 0.91 mm), and SV (67.7 ± 15.2 vs. 57.8 ± 13.9 mL) than nonobese participants (all *p* < 0.05). EF and E/A ratio were similar. BMI correlated positively with EDV and ESV, negatively with E/A ratio, and independently predicted higher EDV and ESV. Obesity is associated with increased LV volumes, septal thickness, and diastolic dysfunction while preserving systolic function, highlighting the importance of early cardiac evaluation in obese adults.

## Graphical abstract

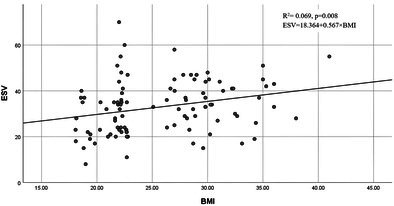

Graphical abstract illustrating the correlation between body mass index (BMI) and left ventricular end‐systolic volume (ESV) in the total study cohort.

## 1 INTRODUCTION

The epidemic of obesity in the last 50 years is a threat to global health, having risen to pandemic levels. It poses a major risk of many chronic illnesses—such as type 2 diabetes, cardiovascular diseases, and specific solid cancers, as well as leading to decreased quality of life, premature deaths, and economic costs (Blüher, 2019). Even though its effects are common knowledge at this point, prevention and treatment are not particularly successful long‐term, partly because physiological processes that defend weight loss are complex.

The hemodynamic and metabolic abnormalities caused by the excessive adiposity cause cardiac remodeling usually presented as left ventricular hypertrophy, alteration of ventricular volumes, and compromised systolic and diastolic performances (Ren et al., 2021). Such changes can occur in a silent manner ahead of the clinical manifestation which raises the risk of development of heart failure and mortality (Abel et al., 2008).

Although the overall adverse effect that obesity causes to the cardiovascular system had been established (Csige et al., 2018; Ortega‐Loubon et al., 2019), the specifics of its effect on left ventricular functioning have yet to be fully described, especially in the populations of the developing world where obesity is on the rise and cardiovascular resources are limited. The majority of the available literature has been carried out among (Western) populations, which limits the generalizability of results to diverse ethnic and demographic backgrounds. Moreover, there is conflicting evidence of the effect of obesity on the major echocardiographic measurements, including EF, end‐systolic volume (ESV), end‐diastolic volume (EDV), and diagnostic indices of diastolic function, including E/A and E/e′ ratios (Peterson et al., 2004; Powell et al., 2006).

Echocardiography is a readily available and noninvasive tool which allows extensive examination of cardiac structure and performance. It offers quantitative measures that are crucial to early detection of subclinical myocardial dysfunction. The relationship between BMI and echocardiographic indicators of cardiac structure and functions adjusted to the confounding variables of age, sex, and blood pressure is crucial in identifying people at risk of developing cardiovascular complications.

The purpose of the present study is to explore the association between body mass index (BMI) and alterations in left ventricular systolic and diastolic functions among adults with and without obesity in our region. We will use multivariable regression analyses to characterize distinct relationships and offer innovative knowledge relating to our population. The study will not only contribute to the knowledge on obesity‐induced cardiac remodeling but also assist the proposed clinical practice to intervene early and offer care in resource‐limited environments.

## 2 METHODOLOGY

This cross‐sectional study was carried out at Sylhet MAG osmani medical college, Sylhet, Bangladesh between January 2024 and December 2024. The protocol of the study was approved by the Institutional Review Board (IRB) of Sylhet MAG osmani medical college, Sylhet, and written informed consent was received from all the research participants that were enrolled in the study.

### 2.1 Participants

One hundred adult subjects at the age of 18 years old and above were enrolled. The participants were divided into two strata according to the BMI based on the World Health Organization criteria like obese (BMI 25 kg/m^2^ and above) and nonobese (BMI less than 25 kg/m^2^), A total of 50 participants were in each stratum. Exclusion criteria were prior known history of cardiovascular disease (e.g., ischemic heart disease and cardiomyopathy), valvular heart disease, congenital heart defects, arrhythmias, uncontrolled hypertension, diabetes mellitus, chronic kidney disease, and any systemic illness that might affect cardiac performance.

Hypertension was defined according to European Society of Hypertension/European Society of Cardiology (ESH/ESC) guidelines 2024 (systolic blood pressure ≥ 140 mmHg and/or diastolic blood pressure ≥ 90 mmHg, or current use of antihypertensive medication) (McEvoy et al., 2024). Participants with uncontrolled (SBP ≥ 140 mmHg or DBP ≥ 90 mmHg despite treatment) hypertension at the time of recruitment were excluded. However, those with controlled hypertension on stable antihypertensive therapy were included, as their inclusion reflects real‐world populations of both obese and nonobese adults with stable cardiovascular profiles. These participants were evenly distributed across the study groups, minimizing potential bias.

### 2.2 Data collection

Face‐to‐face structured interviews were conducted to collect baseline sociodemographic and clinical data. Variables included were age, sex, religion, and residence (urban/rural), level of education, occupation, and blood pressure readings. The blood pressure was measured in a calibrated sphygmomanometer following standardized methods, and their mean of two readings was captured.

### 2.3 Anthropometric measurements

The body weight was taken while wearing light clothes and no shoes on a digital scale. Using a stadiometer, height was measured. BMI was calculated as weight in kilograms divided by the square of height in meters (kg/m^2^).

### 2.4 Echocardiographic assessment

Transthoracic echocardiography was carried out with the help of an Arietta 65 (Fujifilm) by a certified cardiologist who could not know about the BMI status of the participants. The American Society of Echocardiography (ASE) procedures were adhered to in getting the standard two‐dimensional, M‐mode, and Doppler echocardiographic measurements.

Left ventricular end‐diastolic volume (EDV) and end‐systolic volume (ESV) were measured, and ejection fraction (EF) was calculated using the Simpson's biplane method. Diastolic function was evaluated using transmitral Doppler imaging, including early (E) and late (A) diastolic mitral inflow velocities, early diastolic mitral annular velocity (e′), and the E/A and E/e′ ratios.

Interventricular septal thickness in diastole (IVSd) was measured as an index of left ventricular wall thickness. Stroke volume (SV) was derived from left ventricular outflow tract (LVOT) dimensions and velocity–time integral, and cardiac output (CO) was calculated as the product of SV and heart rate. Diastolic dysfunction was graded according to American Society of Echocardiography (ASE) recommendations.

### 2.5 Statistical analysis

Data analyses were conducted using SPSS 25. Normality of continuous variables was initially tested using the Shapiro–Wilk test and by viewing the density of histograms. The data that followed the normal distribution were expressed as and tested with the help of independent samples *t*‐test, and the non‐normal distribution data were tested with the Mann–Whitney *U* test. Categorical data were presented in terms of frequency and percentages, and the difference between groups was tested by Chi‐square or Fisher exact where indicated. The associations among the BMI and echocardiographic parameters were evaluated using Spearman rank correlation, as their distribution was non‐normal. Univariate linear regression was used to determine the potential predictors of cardiac function parameters, where those variables with a *p* value <0.10 and having clinical significance were then entered into multivariable linear regression models. Multicollinearity was checked using variance inflation factors (VIF), all of which were within acceptable limits. The threshold of statistical significance was a two‐sided *p* < 0.05.

## 3 RESULTS

A total of 100 adult participants were included and divided equally in two groups. The mean age of participants was 36.22 ± 7.66 years, without any significant group‐wise difference between them (*p* = 0.242). Gender distribution also didn't differ significantly (*p* = 0.689). The mean BMI of obese and nonobese was 30.51 ± 3.36 and 21.18 ± 1.54, respectively. A higher proportion of urban dwellers was reported in the obese group (70% vs. 40%, *p* = 0.003). A statistically significant difference was observed in the occupational distribution between obese and nonobese individuals (*p* < 0.001). Among the obese group, a higher proportion was service holders (56.0%) compared to the nonobese group (18.0%). A significant difference was observed between education level and obesity status (*p* = 0.001). Obese participants were more likely to have higher educational attainment, with 48% being graduates or above, compared to only 14% among nonobese individuals, whereas lower education levels were more prevalent among nonobese participants (Table 1).

**TABLE 1:** Baseline sociodemographic and clinical characteristics of obese and nonobese adult participants.

Variables	Total (*n* = 100)	Obese (*n* = 50)	Nonobese (*n* = 50)	*p* Value
	Mean ± SD/Number (%)	Mean ± SD/Number (%)	Mean ± SD/Number (%)	
Age (years)	36.22 ± 7.66	37.12 ± 7.09	35.32 ± 8.17	0.24
Sex				
Male	52 (52%)	25 (50%)	27 (54.0%)	0.68
Female	48 (48%)	25 (50%)	23 (46%)	
Height (cm)	158.23 ± 9.85	157.54 ± 9.28	158.92 ± 10.44	0.48
Weight (kg)	64.64 ± 14.48	75.16 ± 11.44	54.13 ± 8.19	<0.001
BMI (kg/m^2^)	25.84 ± 5.36	30.51 ± 3.36	21.18 ± 1.54	<0.001
Religion				
Muslim	83 (83.0)	43 (86.0%)	40 (80.0%)	0.42
Hindu	17 (17.0)	7 (14.0%)	10 (20.0%)	
Residence				
Urban	55 (55%)	35 (70.0%)	20 (40.0%)	0.003
Rural	45 (45%)	15 (30.0%)	30 (60.0%)	
Education				
Illiterate	18 (18%)	6 (12.0%)	12 (24.0%)	0.001
Primary	18 (18%)	11 (22.0%)	7 (14.0%)	
SSC	10 (10%)	4 (8.0%)	6 (12.0%)	
HSC	23 (23%)	5 (10.0%)	18 (36.0%)	
Graduate and above	31 (31%)	24 (48.0%)	7 (14.0%)	
Occupation				
Service holders	37 (37%)	28 (56.0%)	9 (18.0%)	<0.001
Business	7 (7%)	2 (4.0%)	5 (10.0%)	
Others	54 (54%)	20 (40.0%)	36 (72.0%)	
SBP (mmHg)	117.05 ± 6.93	118.40 ± 7.65	115.70 ± 5.89	0.05
DBP (mmHg)	75.50 ± 5.96	76.00 ± 5.80	75.00 ± 6.14	0.40

Among the echocardiographic parameters, obese individuals demonstrated significantly higher end‐diastolic volume (EDV: 100.0 ± 23.3 mL vs. 84.6 ± 25.8 mL, *p* = 0.002) and end‐systolic volume (ESV: 35.6 ± 10.0 mL vs. 30.4 ± 12.5 mL, *p* = 0.023) compared with nonobese participants, while ejection fraction remained similar between groups (*p* = 0.941). Doppler analysis showed that obese participants had higher A‐wave velocity (53.7 ± 12.2 cm/s vs. 46.8 ± 10.0 cm/s, *p* = 0.003), lower Doppler e′ velocity (9.1 ± 3.8 cm/s vs. 10.5 ± 2.9 cm/s, *p* = 0.039), and consequently higher E/e′ ratio (7.83 ± 2.79 vs. 6.25 ± 1.67, *p* = 0.001), suggesting impaired LV relaxation. The E‐wave velocity did not differ significantly between groups (*p* = 0.841). Interventricular septal thickness in diastole (IVSd) was greater among obese subjects (9.33 ± 1.53 mm vs. 8.37 ± 0.91 mm, *p* = 0.0002), indicating concentric remodeling. Stroke volume was significantly higher in the obese group (67.7 ± 15.2 mL vs. 57.8 ± 13.9 mL, *p* = 0.001), whereas cardiac output showed no significant difference (*p* = 0.171). Diastolic dysfunction was evaluated according to American Society of Echocardiography (ASE) criteria. In total, six participants (6%) exhibited Grade I diastolic dysfunction, representing an impaired relaxation pattern. All cases occurred among the obese group, whereas none were observed in the nonobese group. This difference was statistically significant (*p* = 0.027, Fisher's exact test) (Table 2).

**TABLE 2:** Comparison of echocardiographic parameters between obese and nonobese participants.

Variables	Total (*n* = 100)	Obese (*n* = 50)	Nonobese (*n* = 50)	*p* Value
	Mean ± SD/Number (Percentage)	Mean ± SD/Number (Percentage)	Mean ± SD/Number (Percentage)	
ESV (mL)	33.03 ± 11.55	35.64 ± 10.00	30.42 ± 12.48	0.02
EDV (mL)	92.30 ± 25.66	100.00 ± 23.28	84.60 ± 25.83	0.002
EF (%)	64.09 ± 5.55	64.05 ± 5.14	64.13 ± 5.92	0.94
E (cm/s)	63.23 ± 15.20	63.53 ± 14.90	62.92 ± 15.64	0.84
A (cm/s)	50.25 ± 11.63	53.68 ± 12.22	46.81 ± 10.00	0.003
é (cm/s)	9.80 ± 3.40	9.10 ± 3.76	10.50 ± 2.87	0.03
IVSd (mm)	8.85 ± 1.34	9.33 ± 1.52	8.37 ± 0.90	0.0002
SV (mL)	62.75 ± 15.31	67.66 ± 15.22	57.84 ± 13.89	0.001
CO (L/min)	6.39 ± 2.11	6.68 ± 2.37	6.10 ± 1.79	0.17
E/A ratio	1.31 ± 0.37	1.25 ± 0.39	1.38 ± 0.35	0.091
E/é ratio	7.04 ± 2.42	7.83 ± 2.79	6.25 ± 1.67	0.001
Diastolic dysfunction	6 (all Grade‐1)	6	0	0.02

Correlation analysis revealed that there was a significant positive correlation between the value of BMI with ESV (*ρ* = 0.280, *p* = 0.005), EDV (*ρ* = 0.349, *p* < 0.001), and E/e′ ratio (*ρ* = 0.293, *p* = 0.003), and a negative correlation with the E/A ratio (*ρ* = −0.207, *p* = 0.038). No significant correlation was observed with EF (*ρ* = −0.028, *p* = 0.786) (Table 3).

**TABLE 3:** Correlation between BMI and echocardiographic parameters (Spearman's rho).

Echocardiographic parameter	Spearman's ρ	*p*‐value
End‐systolic volume (ESV)	0.280	0.005
End‐diastolic volume (EDV)	0.349	0.00003
Ejection fraction (EF)	−0.028	0.786
E/A Ratio	−0.207	0.038
E/é Ratio	0.293	0.003

**FIGURE 1:**
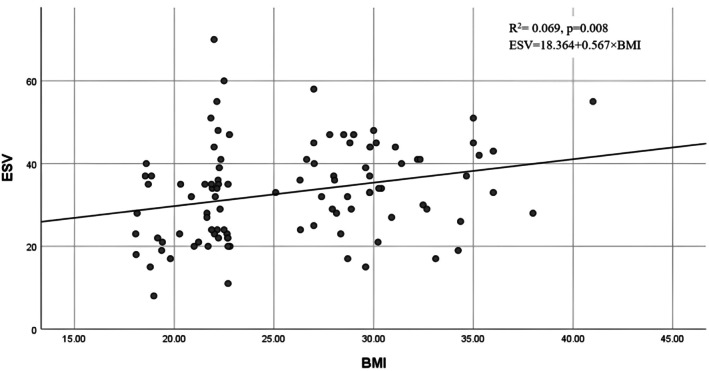
Correlation between BMI and left ventricular end‐systolic volume (ESV) in the total cohort.

**FIGURE 2:**
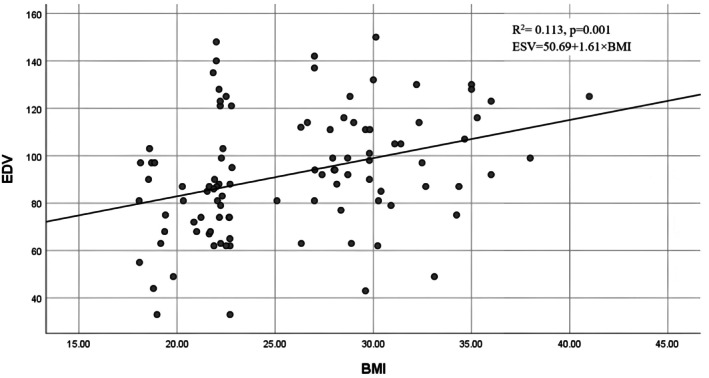
Correlation between BMI and left ventricular end‐diastolic volume (EDV) in the total cohort.

**FIGURE 3:**
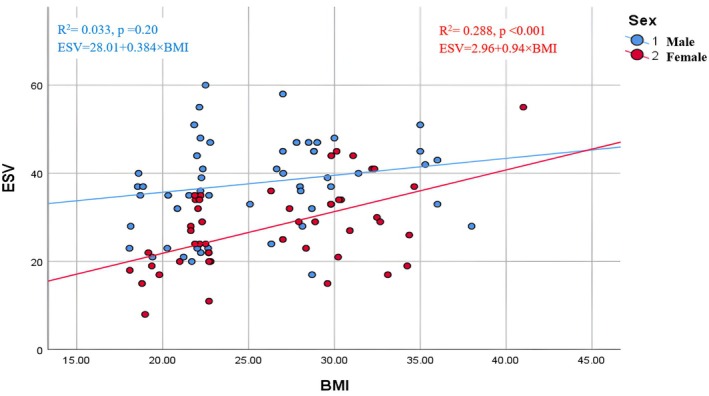
Correlation between BMI and left ventricular end‐systolic volume (ESV) stratified by sex.

**FIGURE 4:**
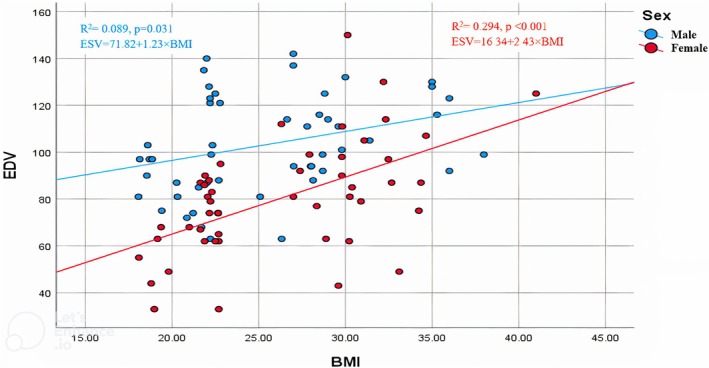
Correlation between BMI and left ventricular end‐diastolic volume (EDV) stratified by sex.

The graphical correlations between BMI and left ventricular volumes are shown in Figures 1, 2, 3, 4. Scatter plots for the whole cohort (Figures 1 and 2) demonstrate positive relationships between BMI and both end‐diastolic (EDV) and end‐systolic volumes (ESV). Separate sex‐specific plots (Figures 3 and 4) with individual regression lines for males and females further confirm similar directional trends across sexes, indicating that the relationship between adiposity and ventricular dilatation is consistent in both groups.

**TABLE 4:** Association of predictors with end‐systolic volume (ESV): Univariate and multivariable linear regression.

Predictor	Univariate *β* (SE)	*p* Value	Multivariable *β* (SE)	*p* Value	95% CI for *β*
BMI	0.567 (0.210)	0.008	0.65 (0.18)	0.001	0.29–1.01
Age	0.47 (0.14)	0.001	0.45 (0.12)	<0.001	0.21–0.70
Sex (F vs. M)	−9.87 (2.10)	<0.001	−10.03 (1.9)	<0.001	−13.81 to −6.26
SBP	−0.24 (0.10)	0.020	−0.48 (0.17)	0.006	−0.82 to −0.14
DBP	0.12 (0.10)	0.240	0.31 (0.20)	0.120	−0.08 to −0.70

Multivariable linear regressions revealed BMI as an independent predictor of elevated ESV (65, *p* = 0.001) and EDV (1.69, *p* < 0.001). Being female was independently linked with decreased ESV and EDV (Table 4 and 5). Advancing age was associated with a significant decrease in left ventricular ejection fraction (EF) (*β* = −0.24, *p* = 0.001), whereas BMI did not have a significant effect (Table 6). The E/A ratio was also negatively predicted by age (*β* = −0.023, *p* < 0.001) (Table 7). Notably, BMI had an independent positive relationship with E/e′ ratio (*β* = 0.118, *p* = 0.011), indicating impaired diastolic function in obese subjects (Table 8).

**TABLE 5:** Univariate and multivariable linear regression of factors associated with end‐diastolic volume (EDV).

Predictor	Univariate *β* (SE)	*p* Value	Multivariable *β* (SE)	*p* Value	95% CI for Multivariable *β*
BMI (kg/m^**2**^)	1.61 (0.45)	0.001	1.69 (0.39)	<0.001	0.91–2.48
Age (years)	0.793 (0.329)	0.01	0.57 (0.27)	0.03	0.03–1.12
Sex (female vs. male)	−24.11 (4.22)	<0.001	−24.131 (4.16)	<0.001	−32.40 to −15.86
SBP (mmHg)	−0.03 (0.37)	0.93	—	—	—
DBP (mmHg)	0.51 (0.43)	0.23	—	—	—

**TABLE 6:** Univariate and multivariable linear regression analysis for predictors of ejection fraction (EF).

Predictor	Univariate *β* (SE)	*p* Value (Univariate)	Multivariable *β* (SE)	*p* Value (Multivariable)	95% CI for Multivariable *β*
Age	−0.22 (0.069)	0.002	−0.244 (0.06)	0.001	−0.38 to −0.10
Sex	1.88 (1.09)	0.08	1.51 (1.06)	0.15	−0.59 to 3.62
BMI	−0.01 (0.10)	0.90	−0.005 (0.10)	0.96	−0.20 to 0.19
SBP	0.06 (0.08)	0.40	0.224 (0.09)	0.02	0.03 to 0.41
DBP	−0.097 (0.09)	0.29	−0.20 (0.11)	0.06	−0.42 to 0.016

**TABLE 7:** Univariate and multivariable linear regression analysis of predictors for E/A ratio.

Predictor	Univariate *β* (SE)	*p* Value	Multivariable *β* (SE)	*p* Value	95% CI for Multivariable *β*
Age	−0.02 (0.004)	<0.001	−0.02 (0.004)	<0.001	−0.03 to −0.014
Sex	−0.08 (0.07)	0.25	−0.08 (0.06)	0.229	−0.22 to 0.05
BMI	−0.01 (0.007)	0.05	−0.007 (0.007)	0.272	−0.02 to 0.006
SBP	−0.007 (0.005)	0.200	−0.003 (0.006)	0.672	−0.015 to 0.010
DBP	−0.002 (0.006)	0.81	0.002 (0.007)	0.775	−0.012 to 0.016

**TABLE 8:** Univariate and multivariable linear regression analyses of predictors of E/é ratio.

Predictor	Univariate *β* (SE)	*p* Value	Multivariable *β* (SE)	*p* Value	95% CI for multivariable *β*
Age	0.05 (0.03)	0.06	0.03 (0.03)	0.21	−0.02 to 0.10
Sex	−0.498 (0.48)	0.30	−0.71 (0.47)	0.13	−1.66 to 0.23
BMI	0.12 (0.04)	0.005	0.11 (0.04)	0.01	0.027 to 0.208
SBP	0.05 (0.03)	0.13	0.05 (0.04)	0.22	−0.03 to 0.13
DBP	0.006 (0.04)	0.88	−0.062 (0.05)	0.21	−0.16 to 0.03

## 4 DISCUSSION

### 4.1 Key results

This cross‐sectional study was conducted to evaluate left ventricular (LV) systolic and diastolic function between obese and nonobese adults by means of transthoracic echocardiography. The results revealed that obese patients had significantly larger left ventricular end‐systolic volume (ESV), end‐diastolic volume (EDV), and E/e ratio, which means that there are larger cardiac volumes and poor diastolic function. EF, however, was not significantly different between the groups. BMI was found to be an independent predictor of ESV, EDV, and E/e ratio. These findings support the hypothesis that obesity is involved in subclinical alterations in cardiac structure and performance.

### 4.2 Interpretation in light of existing literature

This study has revealed that obesity is an important factor of changes in LV structure and diastolic functioning, even in case the subjects have no overt cardiovascular disease. Particularly, BMI was independently linked to elevated end‐systolic volume (ESV) and end‐diastolic volume (EDV), as well as E/e′ ratio, without compromising the EF. These observations contribute to the evidence base that obesity causes early subclinical cardiac remodeling, especially on diastolic performance.

It is worth noting that heart size and volume are known to scale with body size according to established physiological scaling laws rather than linearly. Accordingly, the observed increases in LV volumes among obese participants may reflect both absolute and relative changes associated with body mass. The inclusion of height and weight data allows contextualization of LV volume differences within these scaling principles, suggesting that obesity‐related ventricular enlargement exceeds what would be expected solely from body size scaling.

The graphical correlations between BMI and LV volumes (EDV and ESV) further support the quantitative findings that obesity is associated with ventricular dilatation. The comparable distribution patterns among males and females suggest that the relationship between adiposity and LV structural changes is consistent across sexes. These visual trends align with earlier echocardiographic studies, such as those from the Framingham cohort, which demonstrated that increasing BMI is linked to larger LV chamber dimensions and higher cardiac workload, even in individuals without overt cardiovascular disease.

Our findings are comparable to the previous studies done in other populations. For example, the Framingham Heart Study found a significant relationship between rising BMI and the LV mass and wall thickness and chambers, independent of blood pressure and other risk factors (Lauer et al., 1992; von Jeinsen et al., 2020). Likewise, the current study noted left ventricular morphological and functional changes between obese and their nonobese counterparts. These results emphasize the idea that obesity serves as an independent factor in the development of cardiac remodeling and early myocardial impairment.

Beyond the primary parameters, detailed echocardiographic comparison further substantiated these observations. Obese individuals exhibited significantly higher interventricular septal thickness (IVSd: 9.33 ± 1.53 mm vs. 8.37 ± 0.91 mm; *p* = 0.0002) and greater stroke volume (67.7 ± 15.2 mL vs. 57.8 ± 13.9 mL; *p* = 0.001), reflecting early concentric remodeling and volume adaptation. Despite this, cardiac output and EF remained comparable between groups, indicating preserved systolic function. Tissue Doppler assessment showed lower e′ velocity (9.10 ± 3.76 cm/s vs. 10.50 ± 2.87 cm/s; *p* = 0.039) and higher E/e′ ratio (7.83 ± 2.79 vs. 6.25 ± 1.67; *p* = 0.001) among obese participants, consistent with impaired myocardial relaxation and elevated LV filling pressure. The pattern of increased wall thickness, larger chamber volumes, and diastolic dysfunction with preserved EF aligns closely with early obesity‐related cardiac remodeling and HFpEF phenotype.

Previous research demonstrated that obesity triggers various hemodynamic and neurohormonal alterations, such as an increase in cardiac output, the volume of plasma, as well as cardiac sympathetic activity, which all stimulate ventricular hypertrophy and worsen myocardial relaxation (Alpert et al., 2016, 2018). Advance echocardiographic techniques have also confirmed that although subclinical systolic and diastolic dysfunction occur in obese patients, it can be found even in patients with no signs of cardiovascular disease (Kibar et al., 2013; Tumuklu et al., 2007). Our results confirm this as there was a decrease of transmitral E/A ratio and an increase of LV chamber size in the obese group.

In addition, our observation of a greater E/e ratio in the obese group confirms to the existing echocardiographic studies that obesity is an independent risk factor of abnormal LV relaxation and raised LV filling pressure. Previous articles (Burden et al., 2021; Nielsen et al., 2015; Russo et al., 2011) found the results to be congruent, that the diastolic parameters, such as E/A and E/e ratios, are significantly worse in obese patients than in the normal range. Although the difference between the E/A ratio between the groups did not achieve statistical significance (*p* = 0.091), the increase in E/A ratio in the obese subjects (mean 7.83 2.79 vs. 6.25 1.67; *p* = 0.001) deserves attention. This ratio is thought to be an accurate indicator of diastolic dysfunction as well as increased left atrial pressure indicating early myocardial abnormality even in asymptomatic patients.

According to the available evidences, left ventricular EF tends to be normal in obese patients, even in adults with a BMI higher than 40 indicating preserved global systolic function (Aurigemma et al., 2013). This observation is consistent with invasive and noninvasive studies, including those by Powell et al. (2006), which showed no remarkable relation between BMI and EF. Similar findings were observed in our study. Although left ventricular ejection fraction (LV EF) is the most commonly used measure of global systolic function in clinical practice, it is load‐dependent and may not reliably detect subtle myocardial performance changes. Recent evidence suggests that subclinical LV systolic dysfunction can occur in obesity and may not be fully captured by conventional ejection‐phase indices (Alpert et al., 2016). Advanced echocardiographic techniques such as tissue Doppler imaging and speckle‐tracking echocardiography have demonstrated a progressive decrease in the mitral annular systolic velocity and abnormal strain pattern of myocardial in the presence of obesity even without apparent cardiovascular disease (Barbosa et al., 2011; Talano et al., 2008; Tumuklu et al., 2007). Such results show that, although EF can stay even in the normal range, subclinical systolic dysfunction is becoming more noticeable as BMI rises, which may be facilitated by chronic volume overload, excessive LV mass, and other chronic illnesses like hypertension, diabetes, and coronary artery disease (Alpert et al., 2018).

Notably, the EF was not significantly different between obese and nonobese groups, a finding that was in line with existing studies suggesting that when obesity causes cardiac abnormalities, systolic function tends to maintain its integrity in the early derangements. This preserved EF and accompanying diastolic dysfunction reflects the characteristics of heart failure with preserved ejection fraction (HFpEF) whose burden is on the rise in the obese population.

Modern evidence proves that patients with HFpEF are more prone to be obese than patients with heart failure with reduced ejection fraction (HFrEF) (Aryee et al., 2023; Prenner & Mather, 2017). Obesity results in developing HFpEF due to a complex interplay of mechanisms such as enlargement of plasma volume, changes in the myocardial load, dysregulated diastolic relaxation, systemic inflammation, and metabolic disorders (Obokata et al., 2017). It is found that obese HFpEF patients have a unique hemodynamic and structural pattern that is supported by high biventricular filling pressure, concentric LV remodeling, high index of LV mass, and ventricular dilatation. RV based on preserved EF are all supported by reduced oxygen demands (Basha et al., 2025; Borlaug et al., 2023). This present study contributes to evidence by finding that obesity is a condition where EF is maintained in the face of underlying myocardial dysfunction, and emphasizing there is a requirement of more sensitive measures of early systolic functioning in high‐risk populations.

Our regression models also support the independent correlation of BMI with greater LV volumes and diastolic dysfunction even after the influence of confounding factors like age, sex, and blood pressure are considered. Interestingly, SBP and DBP were not always significant in the multivariable interventions, whereby it is important to note that adiposity and hemodynamic load play dominant roles in this alteration.

Although the pattern of relationships observed in our analysis resembles that presented in the existing evidence, the magnitude of effect, especially the pronounced independent correlation between BMI and E/e′ ratio, outlines additional support to this hypothesis, that BMI is not just a marker of risk, but that it is a factor in itself, leading to early cardiac dysfunction. This is particularly applicable to South Asian groups where cardiometabolic risks are more prevalent at lower BMI than in the Western populations (Abate & Chandalia, 2017; Almulhem et al., 2021).

Overall, our findings are in concordance with the existing literature, reinforcing the concept that obesity‐induced structural and functional cardiac changes can be detected early using echocardiography. These subclinical alterations may precede symptomatic heart failure and represent an important window for preventive interventions targeting weight reduction, lifestyle modification, and early cardiovascular screening.

### 4.3 Strengths of the study

This study has several notable strengths. It provides a comprehensive echocardiographic assessment of both systolic and diastolic left ventricular functions among obese and nonobese adults, encompassing parameters such as ESV, EDV, EF, E/A, E/e′, interventricular septal thickness (IVSd), stroke volume, and cardiac output. The inclusion of diastolic dysfunction grading further refines the functional characterization. Rigorous statistical analyses—including normality testing, bivariate correlations, and multivariable regression with adjustment for key confounders—enhance the robustness of the findings. The use of a well‐matched nonobese comparison group minimizes selection bias and strengthens internal validity. Furthermore, the study contributes novel insights into obesity‐related cardiac remodeling in a South Asian population, a group underrepresented in cardiovascular imaging research, thereby supporting the development of region‐specific clinical strategies.

### 4.4 Limitations

A number of limitations ought to be recognized. Obesity and cardiac changes cannot be determined to be causally related because it is a cross‐sectional study. Statistical power and generalizability may also be limited due to the single‐center setting and the relatively small sample size. Despite adjusting for key confounders, some important factors, such as physical activity levels, overall fitness, and detailed metabolic profiles were not assessed. These unmeasured variables may have influenced the observed cardiac differences between obese and nonobese participants. Although participants with uncontrolled hypertension, diabetes, and chronic kidney disease were excluded, we did not collect detailed biochemical data such as fasting glucose, lipid profile, renal function, or inflammatory markers, which could provide additional insights into subclinical metabolic alterations. In this study, the echocardiographer was blinded to the participants' recorded BMI; it is possible that visual assessment of body habitus could have subtly influenced measurements. This represents a potential source of observer bias.

## AUTHOR CONTRIBUTIONS

Nowrin Kashem, Foysal Ahmed, and Sumona Tanu: Conceptualization, data curation, investigation, methodology, writing—original draft, and writing—review and editing. Md Asaduzzaman: Conceptualization, methodology, formal analysis, project administration, writing—original draft, and writing—review and editing. Anamur Rahman, Rehana Sultana, and Yeasmin Jui: Resources, supervision, writing—review and editing.

## FUNDING INFORMATION

The present study was not funded by any external sources of funding.

## CONFLICT OF INTEREST STATEMENT

The authors do not have any known competing financial interest or relationships that can possibly affect the work reported in this manuscript.

## ETHICS STATEMENT

The ethical approval of this study has been obtained by the institutional ethical review board of Sylhet MAG Osmani Medical College, Sylhet, Bangladesh. This work was carried out following the ethical principles of the Declaration of Helsinki in 1964 and all its amendments or other similar ethical principles. The participants would receive notifications that they are included in the research.

## CONSENT TO PARTICIPATE

Informed consent was written and a record was taken. Participants were reminded that they had the right not to participate or withdraw the consent at any phase of the study. The data analyzed were deidentified.

## Data Availability

All data produced and/or analyzed in the current study is available upon request to the corresponding author.
